# Supplementary material for: Modulation of Macrophage Activity by Pulsed Electromagnetic Fields in the Context of Fracture Healing
Source: Bioengineering (Basel). 2021 Oct 29;8(11):167. doi: 10.3390/bioengineering8110167 (PMC8615107; doi:10.3390/bioengineering8110167)
Supplement: Supplementary file 1 [file bioengineering-08-00167-s001.zip › bioengineering-1422234-supplementary.pdf]

# Modulation of Macrophage Activity by Pulsed Electromagnetic Fields in the Context of Fracture Healing

**Yangmengfan Chen, Maximilian M. Menger, Benedikt J. Braun, Sara Schweizer, Caren Linnemann, Karsten Falldorf, Michael Ronniger, Hongbo Wang, Tina Histing, Andreas K. Nussler and Sabrina Ehnert**

## Uncropped Western blot images for Phospho-Stat1:

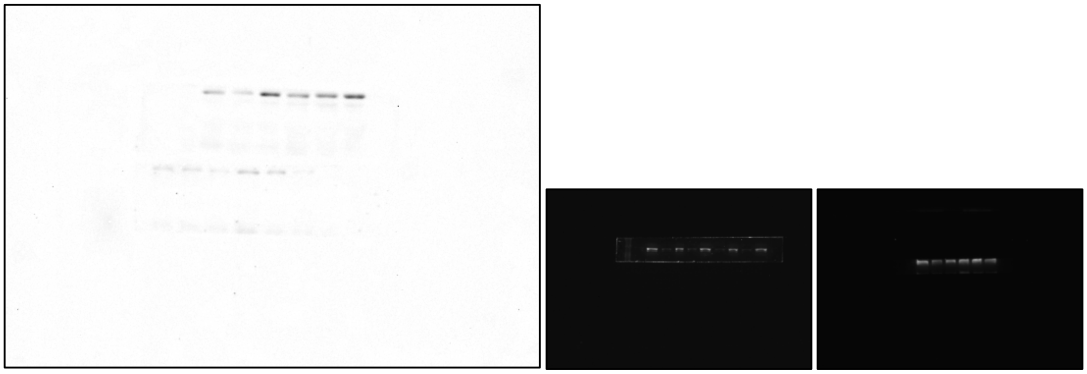

(2 Membranes / invers) other membranes

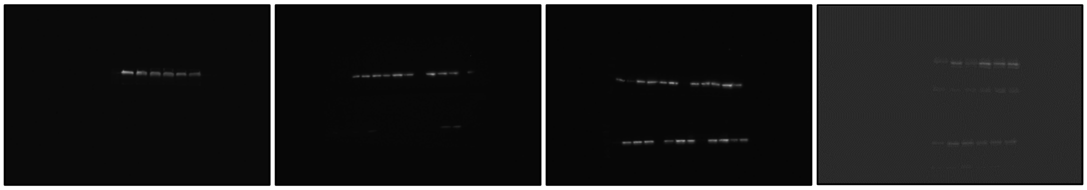

## Uncropped Western blot images for CD86:

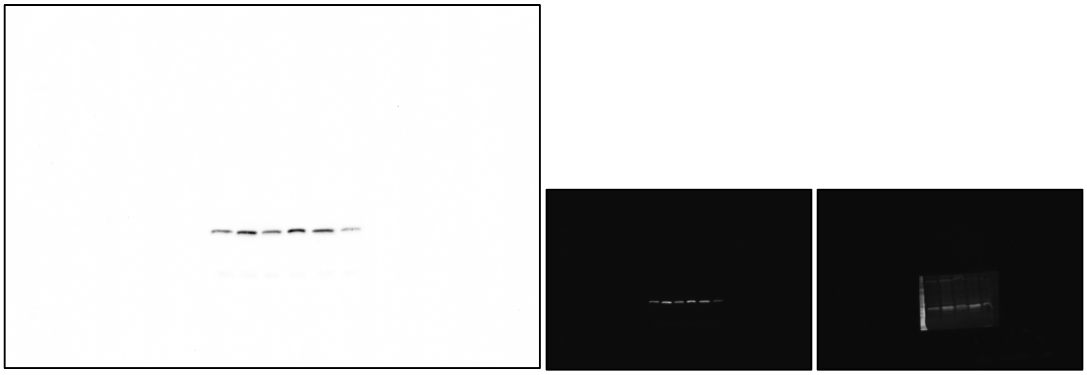

(1 Membrane / invers) other membranes

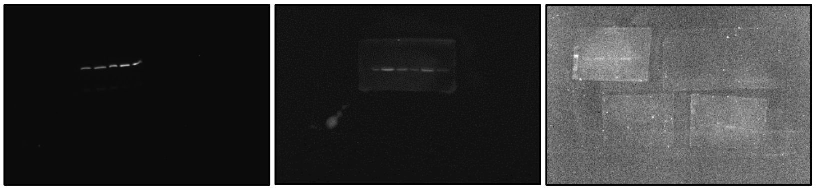

## Uncropped Western blot images for CD86:

## Uncropped Western blot images for Arginase 1:

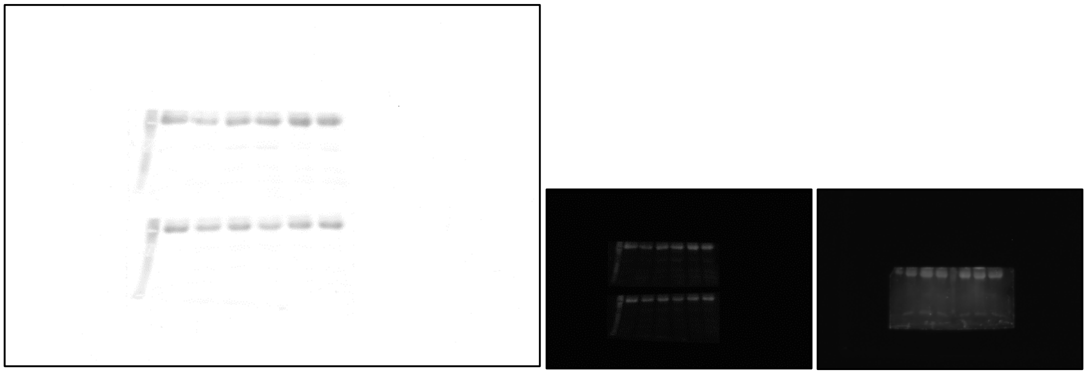

(2 Membranes / invers) other membranes

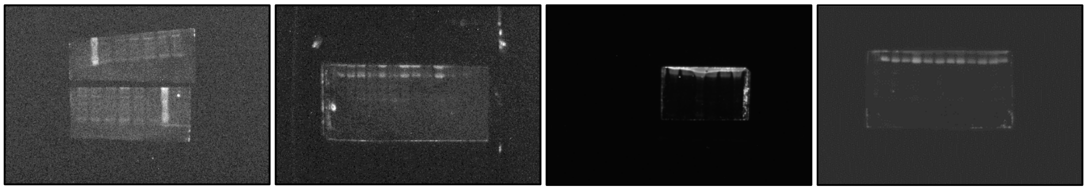

## Uncropped Western blot images for GAPDH:

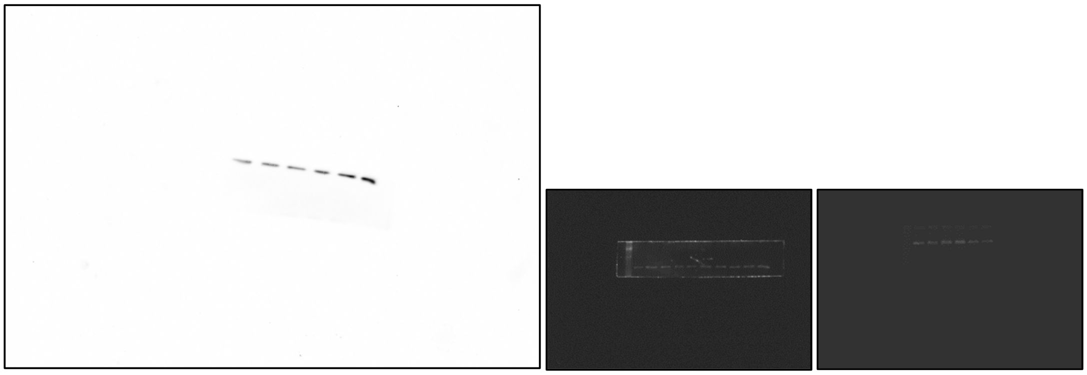

(1 Membrane / invers) other membranes

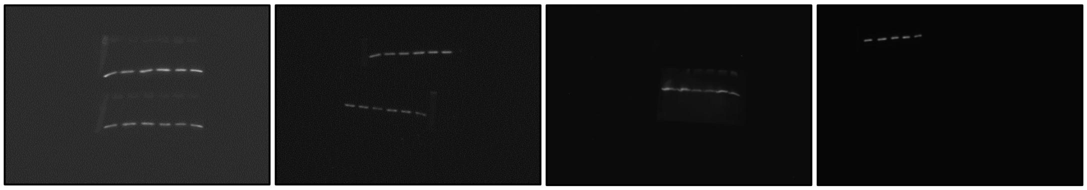

**Figure S1.** Uncropped Western Blot Images. Western blot images shown in Figure 2 A are shown inverse (black on white) as in the manuscript. Other membranes are summarized not inverse.

## Uncropped Agarose Gel Electrophoresis images from all RT-PCRs:

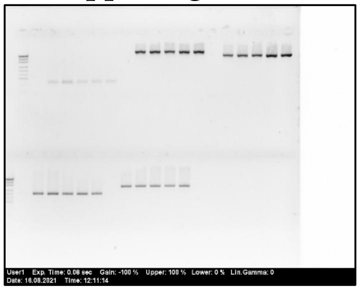

Round 1 n=1

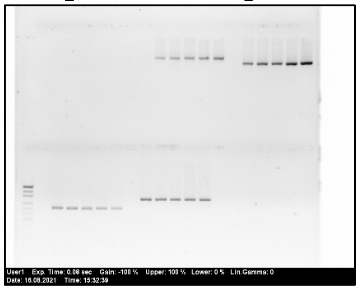

Round 1 n=2

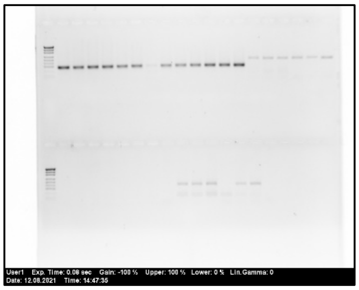

Round 1 RPL13

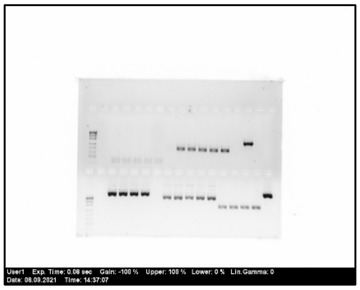

Round 2 n=1

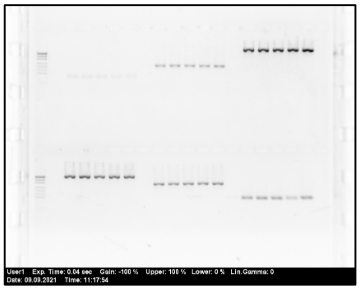

Round 2 n=2

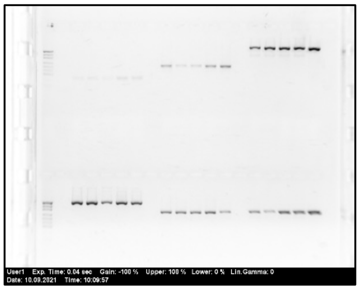

Round 3 n=1

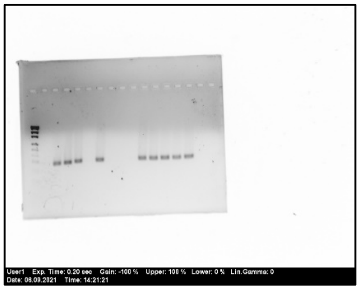

Round 3 RPL13 n=1

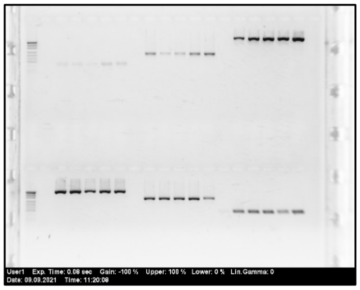

Round 3 n=2

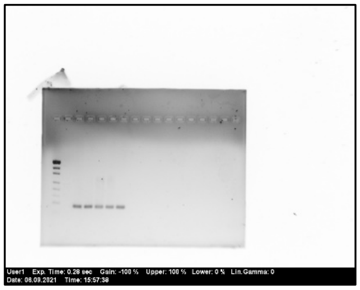

Round 3 RPL13 n=2

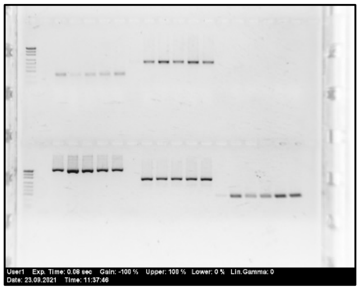

Round 4 n=1

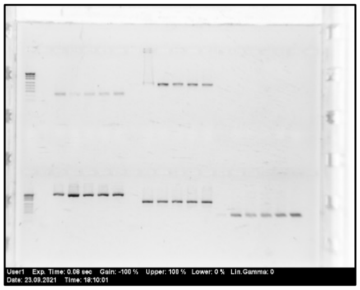

Round 4 n=2

**Figure S2.** Uncropped Agarose Gel Electrophoresis Images shown in Figure 6 A and other rounds. As size marker PUC19 was used. Each round showing bands for Collagen 1A1 (83 bp), RPL13a (100 bp), Fibronectin (203 bp), Versican (306 bp), Biglycan (501 bp), and Decorin genomic DNA (> 500 p).
